# Prospective Heart Tracking for Respiratory Motion Compensation in Whole-heart Magnetic Resonance Angiography

**DOI:** 10.1186/1532-429X-18-S1-P12

**Published:** 2016-01-27

**Authors:** Mehdi Hedjazi Moghari, Tal Geva, Andrew J Powell

**Affiliations:** 1 Cardiology, Boston Children's Hospital, Boston, MA USA; 2 Pediatrics, Harvard Medical School, Boston, MA USA

## Background

Electrocardiogram and respiratory navigator (NAV)-gated 3D whole-heart magnetic resonance angiography (MRA) acquired with an intravascular gadolinium-based contrast agent and a non-selective inversion recovery (IR) pulse to null the myocardial signal generates a high-resolution anatomic dataset allowing for a comprehensive evaluation of intra-cardiac, coronary, and vascular abnormalities [1]. In this technique, an additional IR pulse is also included to selectively restore the signal in the liver, and thus allow NAV tracking of the diaphragm (liver-lung interface). This selective IR pulse, however, excites the blood flowing from veins into the heart creating a bright inflow artifact that hinders image interpretation [2]. Therefore, we sought to develop a prospective respiratory-gating technique (Heart-NAV) that tracks the heart rather than the diaphragm position and eliminates the inflow artifact without compromising image quality.

## Methods

**Figure 1:**
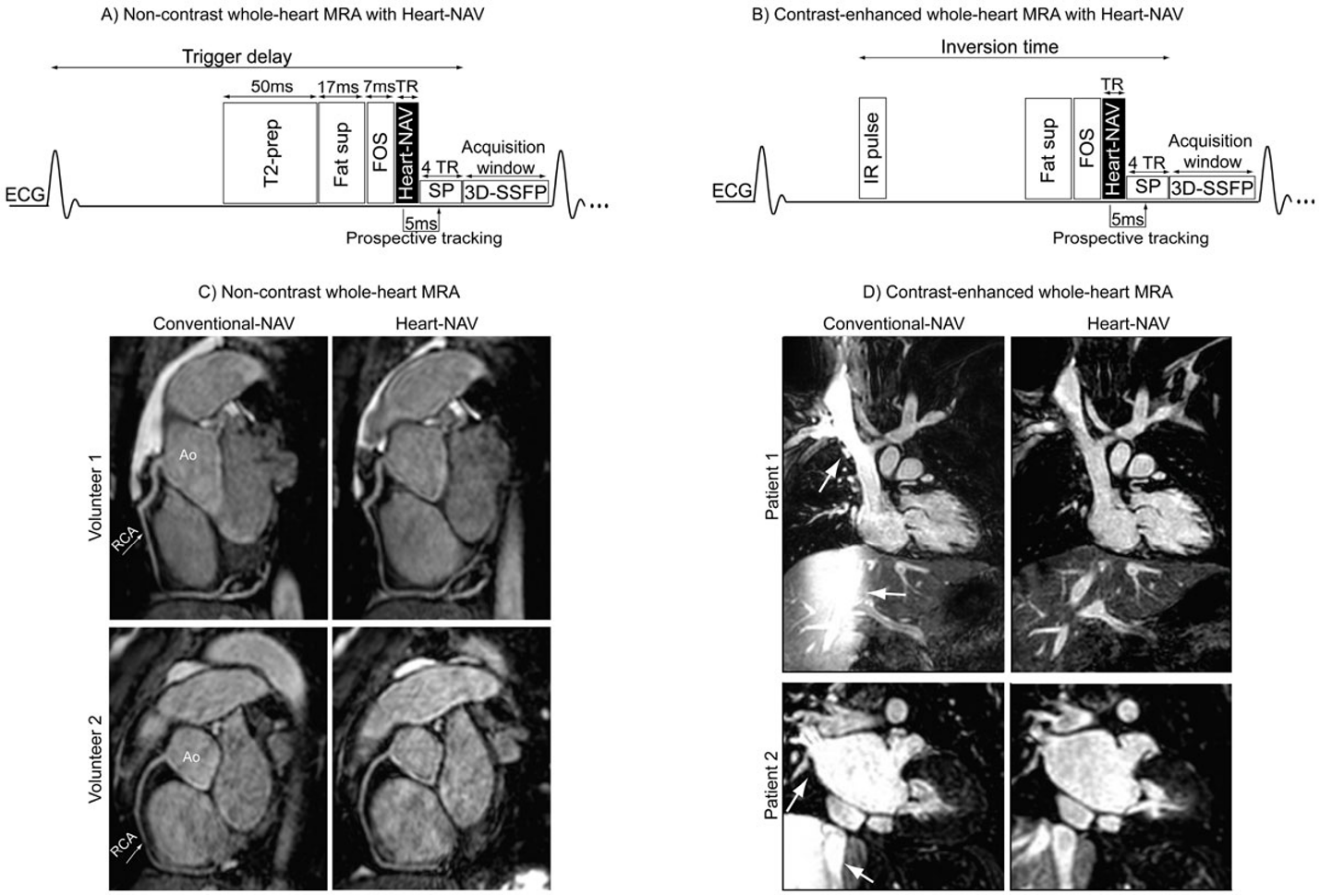
**(A) Schematic diagram of the proposed non-contrast whole-heart MRA acquisition with Heart-NAV**. (B) Schematic diagram of the proposed contrast-enhanced whole-heart MRA with Heart-NAV. (C) Images of non-contrast whole-heart MRA acquisitions with a conventional-NAV and with Heart-NAV from 2 healthy volunteers. (D) Coronal images of contrast-enhanced whole-heart MRA acquisitions with a conventional-NAV and Heart-NAV from 2 patients. Fat sup, fat suppression pulse; FOS, fold-over suppression pulse; IR pulse, inversion recovery pulse; SP, startup pulses; SSFP, steady-state free precession pulse; T2-prep, T2-preparation pulse; TR, repetition time.

Schematics of the proposed Heart-NAV technique for non-contrast and contrast-enhanced MRA sequences are shown in Fig. 1A&1B. One of the startup pulses for MRA sequence is used to collect the centerline of k-space, and its 1-dimensional reconstruction is fed into the conventional-NAV signal analysis process to prospectively gate and track respiratory-induced heart displacement. To assess the efficacy of Heart-NAV in the correction of respiratory motion, 10 volunteers (7 females; age 31 ± 6 years) underwent MRA acquisitions with conventional-NAV and Heart-NAV. For both acquisitions, imaging parameters were FOV ~386 × 230 × 120 mm^3^, spatial resolution 1.5 mm^3^; α/TE/TR 90°/2.4/4.7 ms, bandwidth 0.54 kHz, SENSE factor of 2, acceptance window of 5 mm, and a 32-element phased-array coil. To compare their image quality, sharpness of the coronary arteries was subjectively graded by 2 clinicians and objectively measured (Soap Bubble tool). Subjective and objective measures were compared using a signed-rank test and paired student t-test, respectively. To evaluate the effect on image inflow artifact, 6 patients (4 males; ages 0.3-6 years) each underwent contrast-enhanced (0.03 mmol/kg of gadofosveset trisodium) IR MRA acquisitions with a conventional-NAV and with Heart-NAV.

## Results

All acquisitions were successfully completed. Images from 2 healthy subjects with the non-contrast MRA sequences are shown in Fig. 1C. The vessel sharpness and image quality were equivalent for conventional-NAV and Heart-NAV acquisitions but the imaging time of Heart-NAV was 10% shorter (Table 1). Fig. 1D displays images with contrast-enhanced MRA acquisitions from 2 patients. Inflow artifact was present with the conventional-NAV but not with Heart-NAV.

**Table 1:** Comparison of conventional-NAV and Heart-NAV for non-contrast whole-heart MRA (n = 10).

	Conventional-NAV	Heart-NAV	p-value
Scan time (min)	*8.4 ± 2.2*	*7.5 ± 1.7*	*<0.01*
RCA subjective sharpness	*3.67 ± 0.49*	*3.77 ± 0.37*	*0.42*
RCA objective sharpness	*0.64 ± 0.04*	*0.67 ± 0.04*	*0.18*
LAD subjective sharpness	*3.55 ± 0.51*	*3.53 ± 0.46*	*0.91*
LAD objective sharpness	*0.61 ± 0.07*	*0.60 ± 0.07*	*0.62*
LCX subjective sharpness	*3.47 ± 0.55*	*3.43 ± 0.53*	*0.83*
LCX objective sharpness	*0.56 ± 0.07*	*0.56 ± 0.09*	*0.85*

Values are mean ± standard deviation. Subjective sharpness: 1-poor to 4-excellent. Objective sharpness: 0-blurred to 1-sharp. LAD, left anterior descending coronary artery; LCX, left circumflex coronary artery; RCA, right coronary artery.

## Conclusions

Compared to a conventional-NAV, Heart-NAV achieved similar image quality for non-contrast whole-heart MRA, and eliminated inflow artifact in contrast-enhanced whole-heart MRA.

## References

[1] Makowski MR (2011). Radiology.

[2] Peters DC (2007). Radiology.

